# Written Verb Naming Improves After tDCS Over the Left IFG in Primary Progressive Aphasia

**DOI:** 10.3389/fpsyg.2019.01396

**Published:** 2019-06-12

**Authors:** Amberlynn S. Fenner, Kimberly T. Webster, Bronte N. Ficek, Constantine E. Frangakis, Kyrana Tsapkini

**Affiliations:** ^1^Department of Neurology, Johns Hopkins Medicine, Baltimore, MD, United States; ^2^Department of Otolaryngology-Head and Neck Surgery, Johns Hopkins Medicine, Baltimore, MD, United States; ^3^Department of Psychiatry and Behavioral Sciences, Johns Hopkins Medicine, Baltimore, MD, United States; ^4^Department of Radiology, Johns Hopkins Medicine, Baltimore, MD, United States; ^5^Department of Biostatistics, Johns Hopkins School of Public Health, Baltimore, MD, United States; ^6^Department of Cognitive Science, Johns Hopkins University, Baltimore, MD, United States

**Keywords:** transcranial direct current stimulation (tDCS), primary progressive aphasia (PPA), verb naming, written naming, spelling, neuromodulation, electrical stimulation, inferior frontal gyrus (IFG)

## Abstract

Transcranial direct current stimulation (tDCS), a non-invasive neuromodulation technique, is an effective adjunct to naming treatments in post-stroke aphasia and primary progressive aphasia (PPA). Enhanced performance in oral and written naming and spelling of nouns with tDCS has been quantified in detail, but it is not known whether it is effective for verb treatment in PPA. We addressed the question of whether performance in naming and spelling of verbs can be augmented with anodal tDCS over the left inferior frontal gyrus (IFG). We compared tDCS coupled with oral and written verb naming/spelling treatment with oral and written verb naming/spelling treatment alone. In a double-blind, sham-controlled, crossover design, 11 participants with logopenic or non-fluent variant PPA received approximately 15 consecutive sessions of anodal tDCS and sham over the left IFG coupled with oral and written verb-naming + spelling treatment. Written verb-naming performance improved significantly more for trained verbs in the tDCS than the sham condition. Importantly, tDCS effects generalized to untrained items for written verb naming and were significant even at 2 months post-treatment. We conclude that tDCS over the left IFG can improve written verb naming and spelling in PPA.

## Introduction

Noun and verb naming deficits are common in post-stroke aphasia as well as in primary progressive aphasia (PPA), a neurodegenerative syndrome of variable pathology that primarily affects language functions (Thompson et al., 2012). More specifically, greater deficits in verb naming as compared to noun naming have been documented in non-fluent PPA (nfvPPA) and logopenic PPA (lvPPA) while greater deficits in noun naming have been documented in semantic PPA (svPPA) (Bak and Hodges, 2003; Hillis et al., 2004, 2006; Thompson et al., 2012). In addition to verb naming, verb deficits manifest in other tasks such as verbal fluency and connected speech among those with nfPPA and lvPPA (Davis et al., 2010; Thompson et al., 2012; Hillis, 2013).

Furthermore, we have shown that in PPA naming and writing of single nouns and verbs depends on the retrieval of the lexical form from inferior temporal areas (Race et al., 2013; Riello et al., 2018). Other studies in PPA have shown that syntactic processing of verbs involves frontal and parietal areas of the language network (Thompson et al., 2012). These results converge with evidence from neuroimaging studies on healthy individuals which show that the brain areas involved in verb processing depend on the task being performed and may also be different when a verb is presented as a single word vs. within a sentence (Price, 2000, 2010; Tyler et al., 2004).

Although verb deficits are common in both post-stroke aphasia and PPA, the majority of current studies on language rehabilitation in aphasia, including PPA, specifically target noun rather than verb naming deficits (Tippett et al., 2015). To our knowledge, there have been no attempts to rehabilitate verb naming in PPA using tDCS. The present study covers this gap. In particular, we asked whether verb naming can be improved by tDCS over the left IFG in lvPPA and nfvPPA.

### Challenges in Verb Rehabilitation

Behavioral improvements following speech-language therapy (SLT) for verb production at the single-word level are more challenging to achieve as compared to similar SLT targeting only nouns (Webster and Whitworth, 2012; de Aguiar et al., 2015). The evidence for this may relate to the distinct psycholinguistic dimensions of verbs (de Aguiar et al., 2015; Beber et al., 2018) and depend on the level of impairment (i.e., morphophonological vs. morphosyntactic) (Tsapkini et al., 2002). Nouns describe objects while verbs describe actions, and verbs have an additional feature: they carry information about the structure of an argument, which is essential to sentence production and comprehension. Further, verbs occur less frequently in language, are morphologically and syntactically more complicated, and are less imageable (i.e., the extent to which a word gives rise to a mental image). For example, even the most concrete verbs have lower imageability than concrete nouns (see Vigliocco et al., 2011). Verbs serve as the essential building blocks to phrases and sentences by which their usage supports meaningful, connected speech and comprehension of social/functional dialogue. As a result, impairments in verb naming may also cause deficits in sentence comprehension and retrieval (Thompson et al., 2012). These features make verbs indispensable for communication, and, therefore, verb impairment in PPA may cause more widespread difficulties in communication especially in variants in which verb deficits are more pronounced (i.e., non-fluent and logopenic PPA).

### Verb Rehabilitation Using tDCS

To our knowledge, only four studies have used tDCS to augment verb processing performance (Marangolo et al., 2013, 2018; de Aguiar et al., 2015; Costa et al., 2017). Given the scarcity of the evidence, we review each study separately below. Overall, even in these few studies the areas of stimulation and tasks varied considerably.

Most studies in verb rehabilitation using tDCS involve individuals with post-stroke aphasia. In the first of verb rehabilitation using tDCS, Marangolo et al. (2013), compared the effects of anodal tDCS over frontal (F5) vs. temporal (CP5) regions according to the EEG 10–20 electrode position system (Homan, 1988). They used 5 × 7 cm^2^ electrodes in seven individuals with chronic post-stroke aphasia. Each participant engaged in action naming during anodal tDCS and sham conditions for five consecutive daily sessions for each condition with 6 days intervals. For each condition the authors used different verb sets and measured sustainability of effects at up to a month post all treatments. There were greater improvements in verb naming for items used in the left frontal vs. the left temporal regions or sham; stimulation over the left frontal region induced 15% more gain than stimulation over the temporal region and sham and the authors emphasized the role of the left frontal region for verb lexical retrieval.

In a subsequent study, de Aguiar et al. (2015) found greater effects of tDCS than sham in verb inflectional production in a group of nine participants with chronic post-stroke aphasia using a double-blind crossover design. Therapy focused on production of the correct verb inflectional form in a sentence. In this study, each participant received active tDCS where the anode was placed over different non-lesioned prefrontal/frontal brain areas which were predetermined by MRI scans and the cathode over their homologous areas in the right hemisphere (RH) or the homologous RH Broca’s area. The different brain areas stimulated across participants included: prefrontal cortex anterior and superior to Broca’s area, left superior/middle temporal gyri, left Broca’s area, and left superior/middle frontal gyri. The authors found that tDCS improved correct verb inflections for both trained and untrained verbs, especially in the first period of stimulation.

Marangolo et al. (2018) compared verb naming (from pictures, involving lexical retrieval) to verb generation (from nouns, involving lexical retrieval and competition) in tDCS over the right cerebellum. The authors employed cathodal stimulation over the cerebellum to reduce the inhibition of the Purkinje cells and increase excitability of the left frontal language areas. Twelve individuals with post-stroke aphasia participated in a randomized, crossover, double-blind design in four conditions: right cathodal and sham during a verb generation task and right cathodal and sham during a verb naming task. Stimulation condition effects were tested at the end of each treatment (5 consecutive days) and after 1 week post-treatment. Performance improved after all conditions, but improvement was significantly larger for right cathodal cerebellar stimulation combined with verb generation vs. sham verb generation only. The study emphasizes the role of the cerebellum not in verb production *per se* but in cognitive demands that some tasks of language production may additionally involve, e.g., the verb generation task.

There is only one case study of verb rehabilitation using tDCS in neurodegeneration. In a single case study involving one patient with dementia, Costa et al. (2017) completed 5 days of tDCS over the right parietal cortex with the anode (5 × 7 cm^2^) located between the P6 and CP6 electrode according to the EEG 10–20 electrode position system (Homan, 1988) and the cathode over the contralateral fronto-polar cortex. The authors decided to stimulate this area after three single tDCS sessions over the right parietal cortex, the left parietal cortex and sham, respectively. The right parietal cortex tDCS induced larger improvement in verb picture naming and auditory comprehension tasks. During the subsequent 5 days tDCS treatment, the patient was engaged in a variety of non-specific language tasks such as writing-to-dictation, reading aloud, and repetition of words and non-words. Verb comprehension was the only task that improved significantly after right parietal tDCS and improvement was maintained for 2 weeks post-treatment.

### The Present Study

Recently, our group and others have shown that the use of tDCS may enhance task-specific improvements (e.g., naming or spelling), and may augment therapy gains when applied to specific regions of the brain (Cotelli et al., 2014a; Tsapkini et al., 2014, 2018). In the largest, to our knowledge, tDCS study in PPA our group found that tDCS over the left IFG along with written naming and spelling therapy resulted in larger and longer-lasting improvements compared to sham coupled with the same therapy. Furthermore, the generalization of tDCS gains persisted at 2 months post-treatment (Tsapkini et al., 2018). In the present study, we used the same neural target for tDCS, the left IFG, as in our previous study (Tsapkini et al., 2018) and as in Marangolo et al. (2013) study. We sought to determine whether tDCS over the left IFG in conjunction with oral and written verb naming/spelling therapy may enhance performance on oral and written verb naming more than sham coupled with the same therapy.

## Materials and Methods

### Participants

**FIGURE 1:**
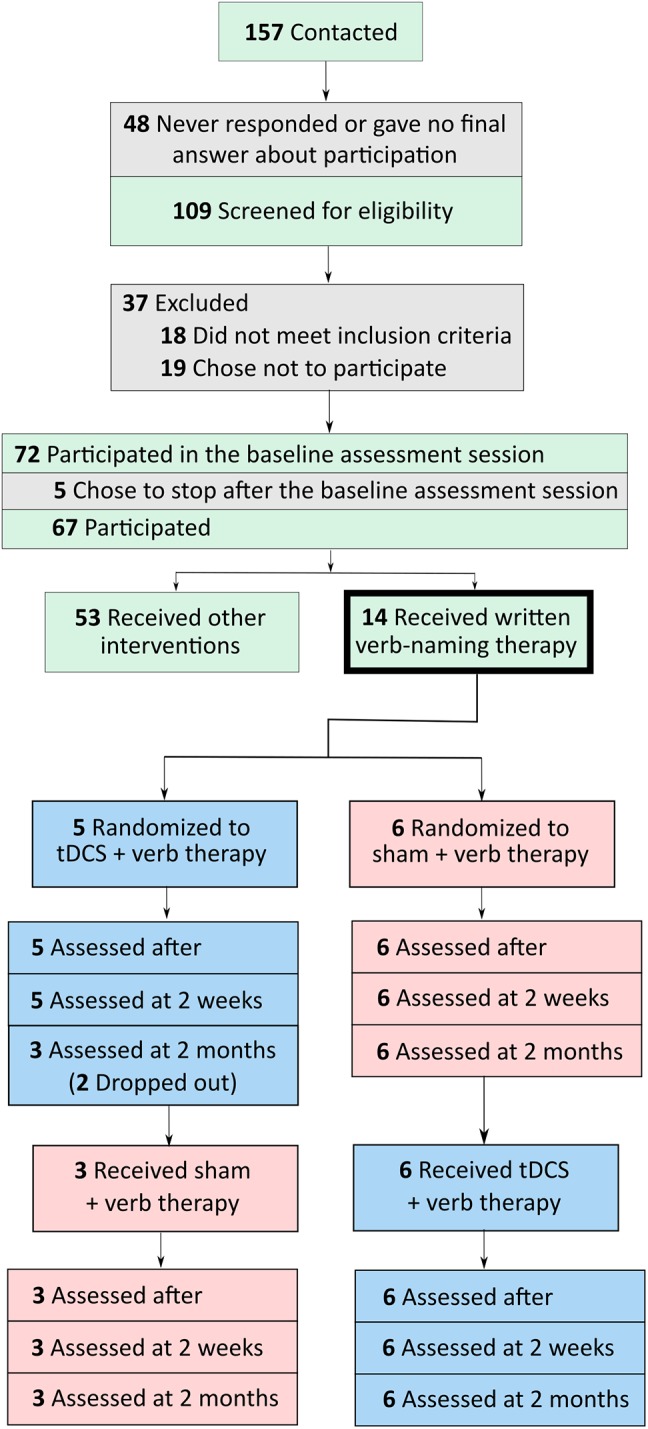
Flow chart of participants, from initial contact to screening to participation in tDCS and sham conditions.

Participants were referred by physicians specializing in PPA and frontotemporal dementia (FTD) from centers around the United States. Referrals were generally based on neurological examination, cognitive-language testing, and neuroimaging measures, including magnetic resonance imaging and positron emission tomography. People were eligible to participate if they were native English speakers with premorbid proficiency in spelling, right-handed, completed a high-school education, and did not have developmental disorders (e.g., dyslexia) or other neurological conditions (e.g., stroke). Participants were recruited through the main clinical trial (ClinicalTrials.gov identifier: NCT02606422). If their performance in noun naming and writing was above criterion (above 80%) but their verb naming was more impaired, then they participated in the present verb naming pilot study. As we showed in a previous study, verb naming deficits are encountered more often in nfvPPA and lvPPA but svPPA are more impaired in noun than verb naming (Riello et al., 2018). Seventy-two participants were recruited in the main trial and 11 participated in the present study, six with nfvPPA and five with lvPPA. A flow chart of participants is shown in Figure 1. Classification of PPA variant was based on consensus criteria reported in Gorno-Tempini et al. (2011). In this double-blind, sham-controlled, crossover-design study, participants were randomly assigned to receive tDCS or sham during the first period of treatment. tDCS and sham groups were, thus, matched for demographic and clinical characteristics, including age, sex, education level, years post onset of symptoms, and language and overall severity measured with the Frontotemporal Dementia Clinical Dementia Rating (FTD-CDR) scale (Knopman et al., 2008). Table 1 presents the demographic and clinical characteristics of each participant.

**Table 1:** Baseline demographics.

	Treatment group	PPA variant	Sessions in period 1	Sessions in period 2	Sex	Years post PPA onset	Age	Language severity	Total severity (FTD-CDR)
P1	t-s	N	12	0	F	6	60	2	8
P2	s-t	L	10	10	M	3.5	69	1	3.5
P3	t-s	N	10	10	F	2	69	2	10
P4	s-t	L	14	13	F	2.5	73	2	7
P5	s-t	N	10	10	M	6	64	3	15
P6	s-t	N	10	10	F	8	66	3	19
P7	t-s	N	12	0	M	2.5	80	2	3
P8	s-t	L	13	12	M	7.5	74	1	3
P9	s-t	L	11	11	M	10	77	2	6.5
P10	t-s	L	10	10	M	4	63	2	9.5
P11	t-s	N	13	12	M	10.5	66	3	6
tDCS mean (s.d.)	5t	4N, 1L	11.4 (1.34)	10.7 (1.15)	2F, 3M	5.0 (3.45)	67.6 (7.70)	2.2 (0.45)	7.3 (2.86)
tDCS mean (s.d.) without P1/P7	3t	2N, 1L	11.0 (1.73)	10.7 (1.15)	1F, 2M	5.5 (4.44)	66.0 (3.00)	2.33 (0.58)	8.5 (2.18)
Sham mean (s.d.)	6s	2N, 4L	11.3 (1.75)	11.0 (1.26)	2F, 4M	6.3 (2.84)	70.5 (5.01)	2.0 (0.89)	9.0 (6.52)
*p*-value (t vs. s, *N* = 11)	–	–	0.945	0.157	–	0.536	0.494	0.644	0.583
*p*-value (t vs. s, *N* = 9)	–	–	0.799	0.710	–	0.808	0.141	0.525	0.870


*Language and total severity are based on scoring from the Frontotemporal Dementia Clinical Dementia Rating scale (Knopman et al., 2008). Means and standard deviations are shown for tDCS-sham and sham-tDCS groups; there were no significant group differences, as detected by a two-tailed t-test. Participants with 0 sessions in period 2 (P1, P7) dropped out of the study after the first period of treatment. tDCS vs. sham comparisons are reported for cohorts with and without P1 and P7. t, tDCS; s, sham; N, non-fluent-variant PPA; L, logopenic-variant PPA.*

### Study Design

**FIGURE 2:**
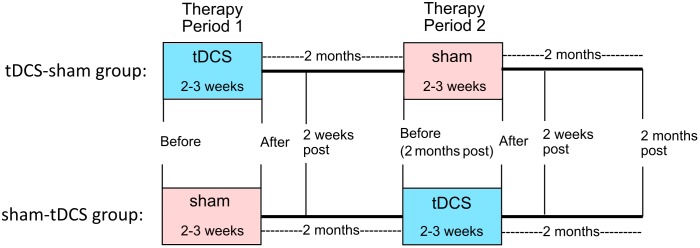
Within-subjects crossover study design. Participants were randomized to receive either tDCS or sham first (tDCS-sham group and sham-tDCS group, respectively). Therapy with active or sham tDCS was given for 2–3 weeks (period 1), followed by 2 months of no treatment. Then therapy with sham or active tDCS was given for 2–3 weeks (period 2). Follow-up assessments occurred 2 weeks and 2 months after the end of periods 1 and 2.

This study was approved by the Johns Hopkins Institutional Review Board. All treatments took place at the Johns Hopkins Hospital. In a within-subjects crossover design, participants received anodal tDCS over the left IFG coupled with verb therapy, and sham stimulation combined with verb therapy. Stratified randomization of stimulation condition within each variant determined whether each participant received tDCS or sham first. The crossover design facilitated recruitment and, importantly for people with PPA, reduced effects of individual variability. Statistical analyses incorporating the crossover design accounted for any neurodegenerative decline over time and potential tDCS carry-over effects. We used the same design as in the main clinical trial (ClinicalTrials.gov identifier: NCT02606422). Figure 2 shows the study design.

Participants received 10–14 consecutive therapy sessions in each treatment period (tDCS mean: 11.43, standard deviation: 1.22, median: 11.5; sham mean: 11.33, standard deviation: 1.37, median: 12) in five sessions per week lasting 40–60 min each. The second period began 2 months after the first period ended. For two participants with other health issues, 4 months separated each period; for two others (tDCS-sham condition), health or behavioral issues prevented them from participating in the second period. Figure 2, 3 show the total number of people included in the analysis at each time point, accounting for the two people who dropped out at the end of period 1. Table 2 presents participants’ performance in language and cognitive tasks at baseline.

**FIGURE 3:**
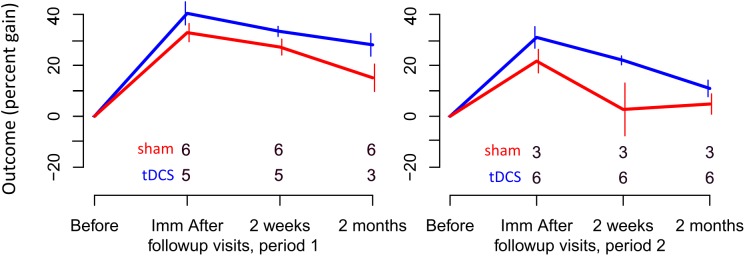
Percent gain in performance for trained items. Top and bottom rows of numbers represent the number of people in the sham and tDCS groups, respectively, showing at which time points two participants dropped out.

**Table 2:** Baseline language and cognitive scores for each participant.

Participant	Letter fluency	Semantic fluency	Digit span forward	Digit Span backward	Spatial span forward	Spatial Span backward	JHU Sentence anagrams active (5 total)	JHU Sentence anagrams passive (5 total)	BNT (30 total)	HANA (35 total)	SOAP: overall (40 total)	SOAP: active (10 total)	SOAP passive (10 total)	SOAP: subject- relative (10 total)	SOAP: object- relative (10 total)
P1	16	10	2	2.5	3.5	4	0	0	10	8	16	7	3	5	1
P2	12	7	5.5	2	2.5	1.5	5	4	16	16	33	8	7	9	9
P3	4	6	3	2	3.5	3.5	4	0	18	14	25	8	5	6	6
P4	9	22	4	2.5	3	3.5	5	2	22	23	31	8	9	6	8
P5	4	12	3.5	1.5	3.5	2.5	0	0	23	14	20	7	6	3	4
P6	9	8	2	2.5	3.5	1.5	2	0	14	15	32	9	9	7	7
P7	17	23	3.5	4	2.5	3.5	5	2	27	29	33	9	10	9	5
P8	17	18	4	3	3.5	3.5	2	1	28	28	27	7	7	7	6
P9	19	11	5	2.5	5	3	5	4	14	16	34	10	9	8	7
P10	8	11	4	2	2.5	1.5	4	0	27	25	21	5	8	4	4
P11	16	17	3.5	0	3	3.5	4	0	25	26	21	7	3	9	2
tDCS mean	12.20	13.40	3.20	2.10	3.00	3.20	3.40	0.40	21.40	20.40	23.20	7.20	5.80	6.60	3.60
(s.d.)	(5.85)	(6.66)	(0.76)	(1.43)	(0.50)	(0.97)	(1.95)	(0.89)	(7.37)	(8.96)	(6.34)	(1.48)	(3.11)	(2.3)	(2.07)
Sham	11.67	13.00	4.00	2.33	3.50	2.58	3.17	1.83	19.50	18.67	29.50	8.17	7.83	6.67	6.83
mean (s.d.)	(5.57)	(5.87)	(1.22)	(0.51)	(0.84)	(0.92)	(2.14)	(1.83)	(5.72)	(5.57)	(5.24)	(1.17)	(1.32)	(2.07)	(1.72)


*Means and standard deviations are shown for tDCS-sham and sham-tDCS groups; there were no significant group differences. Letter and semantic fluency scores are the sum of words starting with F, A, and S and sum of fruits, animals, and vegetables generated in 1 min each. Scores for digit and spatial spans forward and backward are number of successful digits correctly repeated. Johns Hopkins University (JHU) Sentence Anagrams is an in-house task of rearranging words into active and passive sentences. The Subject-relative, Object-relative, Active, and Passive (SOAP) Syntactic Battery tests syntactic comprehension (Love and Oster, 2002). BNT, Boston Naming Test (object naming); HANA, Hopkins Assessment for Naming Actions (action naming).*

Two sets of verbs – trained items (practiced during each session) and untrained items (never practiced but evaluated at follow-up time points in each period of stimulation) – were evaluated before, immediately after, and 2 weeks and 2 months after each treatment period. These two sets of trained and untrained verbs were personalized, and therefore different, for each participant. In addition, the number of words in each trained/untrained set varied within and between participants from 12 to 35 verbs depending on each participant’s severity and progression. Evaluations measured letter accuracy as well as the accuracy of oral production. Participants, technicians administering evaluations, and the speech therapist administering treatment were blinded to treatment condition. The two technicians administering assessments were well-trained by the same neuropsychologist, and the speech-language therapist alone administered treatment.

### tDCS Methods

tDCS was delivered using the Soterix Transcranial Direct Current Stimulator Clinical Trials device, Model 1500. Current was delivered at 2 mA intensity (estimated current density 0.08 mA/cm^2^) for 20 min in the tDCS condition and 30 s in the sham condition, for which current was ramped up and immediately back down to 0 mA at stimulation onset. In both tDCS and sham conditions, participants noted a temporary tingling sensation which lasted for approximately 30 s; this has been known to successfully blind participants to their assigned stimulation condition (Homan, 1988). To reduce the potential for reactions between the skin and electrodes, saline-soaked 5 cm × 5 cm sponges were placed inside non-metallic, conductive rubber electrodes. Once or twice during each session, participants reported general pain levels with the Wong-Baker FACES Pain Rating Scale^1^. Stimulation began at the onset of verb therapy; after the 20 min of tDCS or sham stimulation, verb therapy continued for 20–25 additional minutes.

The anode was placed over the left frontal lobe, corresponding to the F7 electrode in the EEG 10–20 electrode position system (Homan, 1988) and following procedures in Tsapkini et al. (2014, 2018). The reference electrode, the cathode, was placed on each participant’s right cheek. Electrode patches were 5 cm × 5 cm (2.54 cm/inch), covering the entire left IFG (Price, 2000; Purcell et al., 2011a,b; Planton et al., 2013). In addition, the IFG was individually co-registered to pretreatment magnetic resonance imaging scans using a fiducial marker.

### Verb Naming/Writing/Spelling Therapy

We used the same treatment protocol as in our main trial (Tsapkini et al., 2018), i.e., we combined the spell-study-spell procedure (Rapp and Glucroft, 2009) with an oral and written naming paradigm (Beeson and Egnor, 2006). We developed individualized trained and untrained word sets while keeping the same procedures and outcome measures. The participant was shown a verb pictured on the computer, asked to name it orally, and then to write the name. If the patient could not name the verb portrayed (orally or in writing), they were provided with the correct verb and then asked to write it in a spell-study-spell procedure. An example of the treatment is transcribed in Table 3.

**Table 3:** Transcription of a segment of treatment, as the participant was shown a picture of the action “hatch” and was asked to name it.

Clinician: “What is going on in this picture?”
Participant 9: “Eggs?”
Clinician: “What is the chick doing?”
Participant 9: “The chick…uh breaking up?….uh”
Clinician: “What is the word for that?”
Participant 9: “Uh… uh… What is he doing? He’s… uh, he’s coming out.”
Clinician: “It begins with an /h/.”
Participant 9: “Uh…ha…he – not heating.”
Clinician: (Gives two first letters) “h – a”
Participant 9: “He…uh what’s he doing?”
Clinician: (Gives first syllable) “haa.”
Participant 9: “ha…ha…Okay”
Clinician: “Now put it in a sentence and try to fill in the blank, ‘The chick is___?”’
Participant 9: “hacking?”
Clinician: “That’s close…It’s ‘hatching”’
Participant 9: “hatching, hatching…” attempts to spell, “hathing?”
Clinician: (Gives three letters and “ch” sound) “h-a-t… ‘ch”’
Participant 9: (Completes spelling) “hatch…h-a-t-c-h…h-a-t-c-h-i-n-g”
Clinician: “Yes, hatching. Say it 3 times”
Participant 9: “Hatching, hatching, hatching”
Clinician: “Now use it in a sentence, the chick is…”
Participant 9: “The chick is chicking…uh…hatching…”
Clinician: “hatching from the…”
Participant 9: “…from the egg”
Clinician: “Good, hatching from the egg.”


### Stimuli Selection

Stimuli were chosen from a larger pool of words from the verb database of the International Picture Naming Project (IPNP), as normed in Szekely et al. (2004). A pool of approximately 100 verbs was baselined for each participant before each period, and sets of 12–35 trained and untrained verbs were chosen for each participant. The number of verbs assigned to each participant depended on his or her language severity. Participants at baseline were at different stages in their disease progression and, therefore, had varying degrees of language severity (see baseline performance in Table 1. Thus, more progressed participants were assigned smaller numbers of verbs for trained and untrained sets to avoid excessive fatigue during each training session and adherence to the protocol of training all assigned verbs in each session for oral and written naming and spelling. For example, participants who received 12 words were generally slower in naming and writing each word, and in each treatment session could only cover 12 trained words. Participants who received 35 words were generally faster in naming and writing each word, and in each treatment session could cover 35 words. Different matched sets were prepared for each period of stimulation. While all verbs were extracted from the same word-picture bank (Szekely et al., 2004), not all verbs were the same for all participants. The trained and untrained verbs were matched for frequency, length, imageability, and percentage of correct letters spelled before each period for each participant (see Table 4). Outcomes were evaluated at the single word instead of the sentence level; therefore, verb argument structure was not matched for during verb selection.

**Table 4:** Psycholinguistic measures of trained (T) and untrained (UT) words in each period.

Participant	Treatment condition	Period 1				Period 2			
			
		# of T/UT words	freq_bnc	Length	img	# of T/UT words	freq_bnc	Length	img
P1	t-s	20/20	0.13	0.30	0.97		na^∗^		
P2	s-t	20/20	0.18	0.41	0.16	20/19	0.04	0.55	0.36
P3	t-s	23/24	0.35	0.20	0.51	25/25	0.99	0.38	0.37
P4	s-t	20/20	0.71	0.07	0.16	20/20	0.35	0.33	0.27
P5	s-t	12/14	0.06	0.42	0.96	15/16	0.84	0.11	0.46
P6	s-t	21/20	0.95	0.95	0.29	20/20	0.22	0.62	0.78
P7	t-s	18/18	0.69	0.58	0.31		na^∗^		
P8	s-t	30/30	0.34	0.21	0.33	32/32	0.45	0.64	0.01
P9	s-t	22/25	0.28	0.06	0.43	20/25	0.84	0.81	0.26
P10	t-s	15/16	0.05	0.64	0.21	26/25	0.94	0.30	0.07
P11	t-s	11/17	0.08	0.30	0.07	15/15	0.42	0.32	0.23


*P1–11 represent each individual participant. A two-tailed t-test was done to compare psycholinguistic measures for trained and untrained items within each phase for each individual person. P-values representing statistical difference between trained and untrained items are shown for frequency (British National Corpus (BNC); http://www.natcorp.ox.ac.uk), length, and imageability (img). Participants marked with na^∗^ dropped out after period 1. t, tDCS; s, sham.*

### Calculation of Written Naming/Spelling Scores

Follow-up evaluations occurred immediately after, 2 weeks post-, and 2 months post-treatment in each period. The primary outcome measure, letter accuracy, was calculated, taking into account letter additions, deletions, substitutions, and movements based on the rule-based scoring system developed at JHU (Goodman and Caramazza, 1985). With this rule-based scoring system, each error subtracted one point from the total letters possible for each word. Grossly, each letter received 0.5 points for identity and 0.5 points for correct position. For example, *sing* would have a total possible score of 4 points. Spelled *snig* the score would be 3 out of 4 points for a transposition of letters; the same can be calculated by subtracting 0.5 for wrong positions for each of the letters *n* and *i*. When spelled *senk*, the score would be 2 out of 4 points for two letter substitutions or 1 for the wrong identity for each of the letters *e* and *k*. Total letters correct were summed for all words; this was divided by the total number of possible correct letters for all words. If a participant did not correctly spontaneously name the verb, a score of zero was given for that word, regardless of the number of total letters in the word. The number of verbs written entirely correctly without errors varied by person. The grapheme-by-grapheme scoring system has been shown to be more sensitive than scoring word-by-word; it also captures the error types. Inter-rater reliability was 95%, and consensus scores were generated if discrepancies existed. We have made available the scoring system we used in the Supplementary Materials.

### Statistical Analysis

Statistical analyses are described in detail in Tsapkini et al. (2018). To determine whether there was any additional gain in written verb naming scores for tDCS vs. sham, we used the Generalized Estimating Equation (GEE) approach to estimate the tDCS vs. sham effect at each time point (Liang and Zeger, 1986). Data used for each patient (*i*) were: The order of treatments (order_i_ = TS if patient *i* was treated with tDCS in period one and sham in period two; order_i_ = ST for the reverse order). The change in word production performance immediately after minus before sham (δ*Y*_i__,sham,after_); 2 weeks after minus before sham (δ*Y*_i__,sham,2w_); 2 months after minus before sham (δ*Y*_i__,sham,2m_); and the analogous changes under tDCS (δ*Y*_i__,tDCS,after_; δ*Y*_i__,tDCS,2w_; and δ*Y*_i__,tDCS,2m_, respectively). We analyzed the data at each time point (order_i_, δ*Y*_i__,sham,after_; δ*Y*_i__,tDCS,after_) to estimate the parameters of the standard crossover formulation (Jones and Kenward, 2003). This formulation decomposes the average changes under sham and tDCS into parameters for a treatment effect, a period effect, and a treatment-by-period interaction.

For each follow-up time, we calculated the predictive accuracy by the leave-one-out cross-validated R-squared (Hastie et al., 2009) for (1) the model with a tDCS vs. sham effect alone, (2) the model allowing for an additional period effect, and (3) period the model allowing for an additional interaction effect. The results show the estimates of the tDCS vs. sham effect for the best model at each follow-up time based on the GEE approach (Liang and Zeger, 1986). Significance levels were obtained by comparing the distribution from the permutation of the order-of-treatment assignment (tDCS first, sham second) across patients (Rosenbaum, 1984).

The two people who did not participate in period two were excluded only for the time points in which they did not participate. The total number of participants included at each time point are shown in Figure 1, 2. For each time point of each period, all data possible are included in the calculations of gain, effect size, standard error, and the *p*-value. For each period and time, available data at each period and time are calibrated using a propensity score weight, so that the (weighted) average of their baseline scores be as close as possible to the overall (unweighted) average of baseline scores at the beginning of the study. We did this so that results across periods and times have the same reference set of participants, which would not be possible if participants are entirely ignored when some values are missing.

## Results

### Tolerability

Active tDCS was well-tolerated with no adverse effects and no complaints beyond itching or tingling. Participants rated their pain levels before and during each session on a scale of 0–10 with the Wong-Baker FACES Pain Rating Scale^2^. Mean pain level did not differ between tDCS and sham conditions (tDCS mean 1.49; sham mean 2.29).

### Written Naming Performance in tDCS vs. Sham

Written-naming/spelling scores were calculated as letter accuracy of verbs spontaneously named without a cue, before each period and at each follow-up time point, according to Goodman and Caramazza (1985), as explained above (see also Supplementary Materials for details). Raw scores (percentages) for each individual by time point in each period are reported in Table 5.

**Table 5:** **(A)** Trained items; **(B)** untrained items.

			Period 1				Period 2			
				
Participant	Variant	First-period treatment condition	Before	After	2 weeks	2 months	Before	After	2 weeks	2 months
**(A)**		
P2	L	s	50.33	70.20	81.79	80.46	43.48	82.61	66.46	54.04
P4	L	s	51.66	96.82	66.67	83.57	55.76	97.45	88.50	70.00
P5	N	s	31.25	66.07	57.50	40.74	33.13	79.82	47.37	49.56
P6	N	s	41.61	73.67	71.01	44.97	18.40	48.47	35.58	7.06
P8	L	s	45.59	97.48	93.70	82.13	68.88	98.77	85.89	75.52
P9	L	s	55.79	83.88	79.49	62.27	55.62	78.71	78.92	70.88
		Mean	46.04	81.35	75.02	65.69	45.88	80.97	67.12	54.51
P1	N	t	29.47	88.08	59.60					
P3	N	t	57.38	100.00	93.12	94.57	66.67	99.45	90.50	79.23
P7	N	t	65.93	94.81	93.33					
P10	L	t	61.54	96.40	94.59	90.09	68.33	82.92	72.50	64.17
P11	N	t	59.84	96.77	100.00	78.21	82.46	100.00	62.50	88.46
		Mean	54.83	95.21	88.13	87.62	72.49	94.12	75.17	77.29
**(B)**		
P2	L	s	50.00	51.41	74.65	48.61	69.68	75.48	54.84	59.68
P4	L	s	73.05	70.00	80.53	80.67	59.54	77.50	51.22	70.00
P5	N	s	69.33	73.46	68.24	51.97	48.21	34.78	18.18	50.00
P6	N	s	49.38	51.24	57.14	41.61	41.61	37.27	32.30	5.59
P8	L	s	56.19	61.16	69.91	75.00	74.59	80.89	85.77	92.89
P9	L	s	53.55	59.72	61.70	53.94	53.18	53.44	57.63	63.87
		Mean	58.58	61.16	68.70	58.64	57.80	59.89	49.99	57.00
P1	N	t	16.88	34.97	26.60					
P3	N	t	42.78	56.94	69.44	75.00	56.84	71.84	66.84	65.79
P7	N	t	68.09	78.72	58.16					
P10	L	t	56.30	67.86	84.03	74.79	65.74	79.17	72.69	57.41
P11	N	t	36.36	62.98	69.38	69.70	66.39	57.14	49.50	31.93
		Mean	44.08	60.29	61.52	73.16	62.99	69.38	63.01	51.71


*Raw scores (percentages) are reported for each time point in each period, by participant, grouped by tDCS-sham and sham-tDCS condition. B. Untrained items. Raw scores (percentages) are reported for each time point in each period, by participant, grouped by tDCS-sham and sham-tDCS condition. t, tDCS; s, sham; N, non-fluent PPA; L, logopenic PPA.*

For trained verbs, written naming scores improved significantly more in the tDCS than sham condition immediately after treatment (additional tDCS gain 8.3%, see Table 6 and Figure 3). Gains did not sustain until 2 weeks or 2 months. Gains were similar in both periods, and the best model selected for the trained data was the cumulative model representing both periods.

**Table 6:** Trained items.

Periods 1 and 2: treatment and period model	After	2 weeks post	2 months post
Additional gain (tDCS vs. sham)	8.3	11.7	9.5
Standard error	3.4	5.6	4.3
*p*-value	0.0500	0.0750	0.200


*The best model chosen combined both periods in the treatment and period model.*

**FIGURE 4:**
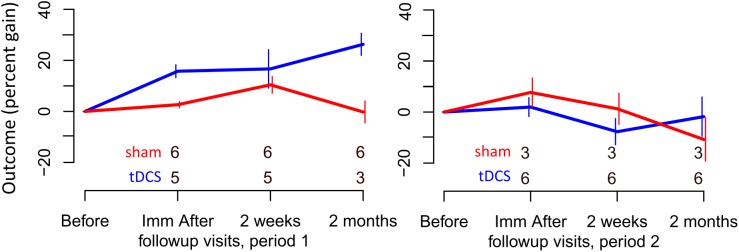
Percent gain in performance for untrained items. Top and bottom rows of numbers represent the number of people in the sham and tDCS groups, respectively, showing at which time points two participants dropped out.

For untrained verbs, written naming scores improved significantly more in the tDCS than sham condition immediately after treatment (additional tDCS gain 13.1%) and at 2 months post-treatment (additional tDCS gain 17.7%, see Table 7 and Figure 4). There was a treatment by period interaction immediately after treatment, indicating that tDCS was more effective immediately after treatment after the first period only. The 2 months tDCS gain was not significantly different between periods 1 and 2 because the combined model was selected. Figures combine data for periods one and two in Supplementary Figures 1, 2.

**Table 7:** Untrained items.

	After	^∗^2 weeks post	^∗^2 months post
Period 1: additional gain (tDCS vs. sham)	13.1	-0.2	17.7
Standard error	2.8	5.3	6
*p*-value	0.000	0.985	0.0250
Period 2: additional gain (tDCS vs. sham)	-5.7		
Standard error	6.8		
*p*-value	0.530		


*Immediately after treatment, the best model chosen involved the interaction of treatment and period. ^∗^Best model chosen combined both periods in the treatment and period model.*

### Exploratory Analyses of Oral Naming

Oral naming results are not presented in the present report; fewer data were available for oral naming analysis due to 2 patient dropouts in the second period and 1 case of mutism. Further, at the time of writing the present manuscript, data were scored using a relatively crude whole-word naming procedure and not at the level of phoneme, that would have been equivalent to the letter accuracy analysis we performed for the written naming data. Given these limitations, we performed an exploratory analysis of the oral naming data available from 10/11 participants pre- and post-therapy at period 1, and 8/11 participants at period 2.

For trained verbs, oral naming scores (in contrast to written naming) did not improve significantly more in the tDCS than sham condition immediately after treatment (additional tDCS gain: 4.6%; standard error: 5.4; p = 0.455). No other significant gains were observed in trained verbs at 2 weeks and 2 months.

For untrained verbs, oral naming scores (similarly to written naming) improved significantly more in the tDCS than sham condition immediately after treatment in period 1 (additional tDCS gain, 14.1; standard error, 2.1; *p* < 0.001). No other significant gains were observed in untrained verbs at 2 weeks and 2 months.

## Discussion

The present study shows for the first time that tDCS over the left IFG improves written verb naming significantly more than sham in lvPPA and nfvPPA. In particular, in a montage where the anode is placed in the frontal operculum to target the left IFG and the cathode on the right cheek, active tDCS augmented written naming of trained verbs (measured by letter accuracy) more than sham immediately after therapy; the gain was significant at 2 weeks and 2 months post-treatment. Furthermore, active tDCS induced larger generalization of therapy gains to untrained verbs. Most importantly, the advantage in untrained verbs was significant even at 2 months post-treatment.

The effects of tDCS over the left IFG on verbs were more attenuated in comparison to the main trial results (Tsapkini et al., 2018) as well as oral noun naming (Cotelli et al., 2014b). For example, the tDCS effect on trained verbs was smaller (8% gain) than in our main study (15% gain) and did not sustain up to 2 months post-treatment. We postulate that this may be due to the differences in these two word classes. Verbs, except in semantic and lexical form (phonological or orthographic), carry rich information about an event, an agent, a recipient of the action (if any) and its timing. Therefore, verbs may be more challenging to treat than nouns as found in a previous tDCS study of verb rehabilitation in post-stroke aphasia (de Aguiar et al., 2015). In addition, we treated only one aspect of verbs: the retrieval of their lexical form but all the other information that is specific to verbs, and particularly their role in a sentence, were not targeted in the present therapy. Given the significant correlation between verb naming and verb processing in sentences (Thompson et al., 2012), future studies should incorporate the sentence level in rehabilitation.

How does the stimulation of the left IFG affect written verb naming and spelling, i.e., the retrieval of the orthographic representations of verbs? On one hand, the left IFG has been traditionally regarded as an area involved in verb processing, although to different degrees depending on the requirements of each task, e.g., single verb naming vs. sentence processing (Tyler et al., 2004; Price, 2010). The left IFG triangularis, in particular, and the inferior temporal gyrus are areas we found to significantly predict verb naming as they predict noun naming (Riello et al., 2018). The left ITG, being an orthographic lexicon substrate as we had previously claimed (Tsapkini and Rapp, 2010), has also been claimed to be a substrate for naming in general (Price and Devlin, 2011). Therefore, if the neural substrates of lexical retrieval of nouns partly overlap with those of verbs and the area stimulated is an area of active retrieval (left IFG triangularis) from the lexical storage of words (left ITG) then it is not surprising that some effects for verbs may be similar with those for nouns. In a previous study, we showed that the tDCS effect is modulated by changes in functional connectivity as measured in resting-state fMRI. In particular, we showed that the increase in written naming/spelling performance for nouns as measured by letter accuracy is correlated with a decrease in correlation between the left IFG triangularis and the middle and inferior temporal areas (Ficek et al., 2018). Although we do not present imaging evidence here for verbs, given the similar behavioral effect, we may speculate that the tDCS effects on verbs involve a similar mechanism.

### Differential Effects of tDCS on Trained and Untrained Items?

In the present study, the additional gain after tDCS compared to sham was greater in the untrained verbs compared to trained verbs. Differential effects of tDCS in trained vs. untrained items (and tasks) have been found in other tDCS in PPA studies, and several times larger improvement in untrained items or tasks than in trained. This is a common finding in previous studies (Cotelli et al., 2014b; Roncero et al., 2017). Cotelli et al. (2014b) found generalization of tDCS effects to untrained tasks, i.e., improvement in naming and daily living language abilities in the Italian adaptation of the Aachen Aphasia Test (Luzzatti et al., 1994), both immediately post and at 12 weeks follow-up time point only for the tDCS group (8/16 patients). Also, Roncero et al. (2017) found generalization of tDCS effects in untrained items in picture naming and untrained digit span for the tDCS condition only (11 patients). Along with these studies, we have also found (Tsapkini et al., 2018) generalization to other items in written naming and spelling in untrained items for the tDCS condition only (36 patients).

We would like to put forth an overall interpretation of these effects. We hypothesized that the effects may be attributable to different mechanisms, i.e., different language and cognitive variables and probably different neural substrates may be associated with the effect of tDCS in trained vs. untrained stimuli. We, thus, performed two separate analyses on the trained and the untrained data of the behavioral and neural predictors of tDCS effects on the main trial data in which we had more power (Tsapkini et al., 2018). We found that different language and cognitive tasks as well as different brain areas predicted tDCS effects in trained vs. untrained Although we cannot perform such an analysis here due to the small number of participants, in the large trial we found that the language and cognitive functions that predict performance in trained items at each follow-up point (immediately post-treatment and at 2 weeks and 2 months post-treatment) were disease progression, learning ability (as evidenced by Rey Auditory Verbal Learning Test, RAVLT) and working memory capacity as evidenced by digit and backward spatial spans. Importantly, these predictors were not different in the tDCS vs. sham (written naming and spelling therapy).

In contrast, for untrained items, the variables that predicted tDCS effects were rule-based learning (as evidenced in letter-sound correspondence ability in pseudoword spelling) and verbal short-term memory (as evidenced in RAVLT). Most importantly, tDCS was the most significant predictor that was associated with performance in untrained words at the 2 months follow-up. Therefore, training effects and generalization effects may be attributable to different language and cognitive mechanisms, as we claim in that study (de Aguiar et al., unpublished).

Additionally, we asked the question of whether there were different brain areas associated with effects in trained and untrained items. We found that, in addition to the overall brain atrophy and baseline performance, the augmentative effect of tDCS over the left IFG on training was associated with the volume of the left angular gyrus, whereas the augmentative effect of tDCS on generalization was associated with the volumes of the left supramarginal gyrus and left dorsolateral prefrontal cortex. The importance of the latter area has also been shown in recent studies where tDCS over this area has resulted in the generalization of tDCS effects in untrained items in naming (Cotelli et al., 2014b).

### Detection of Carryover and Order tDCS Effects in Crossover Designs

A noteworthy finding was that tDCS effects compared to sham were more pronounced in the first period. When the study started the most extended effect for the sustainability of tDCS effects mentioned in the literature was 1 month. We had extended it to 2 months, making a large assumption that the tDCS effect will have washed out by that time. To allow for the case that it does not, we also used the gain as a main outcome, i.e., the score at each follow-up minus the score at baseline of the respective period. This means that any effect of tDCS vs. sham found in period 2 will not reflect the improvement itself at the level of performance in written naming/spelling, i.e., the fact that the patients who got tDCS first are writing more accurately than those who got sham. Therefore, any effect of tDCS vs. sham that carries over from 2 months of period 1 to only *the level of performance in* period 2 will cancel in the tDCS vs. sham comparisons of *gains* at period 2. In this way, we eliminated one of the possible “carryover” effects of tDCS: the improvement at the mere level of performance. However, “carryover” effects of tDCS from period 1 may also reflect other changes, such as a change in the rate of decline in written naming/spelling.

To conclude, diminishing or differential effects of tDCS vs. sham in period 2 (even after removing the effect of tDCS on the mere level of performance as explained above) may be due to, at least, two different reasons. First, the patients declined more at the beginning of the second period, and therefore tDCS may not have been as effective as in the first period. Secondly, tDCS (or sham) in period 1 might affect not just baseline performance in period 2, but also more complex and dynamic processes in neurodegeneration, such as the rate of decline in written naming/spelling. Lastly, a combination of the previous two points may account for the variable effects of tDCS vs. sham in period 2. Therefore, measuring the carryover effect and how the administration of tDCS in the first period affects the second period (order effect) are complicated issues. Disentangling the above possibilities requires further research.

### Limitations: Follow-Up Time Points and Missing Data

We would like to comment on an issue that concerns the 2 weeks follow-up assessment: the additional gain of tDCS for untrained verbs (and performed only in Phase 1) was significant immediately after tDCS treatment and after 2 months but not at 2 weeks post-treatment (*p* = 0.985). We had implemented the 2 weeks follow-up as an early check in case tDCS effects wash out completely. However, it proved to be hard to bring all participants back to Johns Hopkins since many of them had just returned home (many times out of State) and they were finally adjusting to their routine again. Therefore, for 5 out of 11 participants, the 2 weeks evaluation was performed via video-conference. The difference in assessment setting, the possible lack of easiness with the new technology, the artificial and unfamiliar way of assessment may have induced a large variability in this small cohort that canceled out the effects.

The other limitation of the present study is the fact that two patients did not undergo period 2 of stimulations and the number of participants in that period became very small. In patients with a neurodegenerative disease and especially with not very common conditions such as PPA, it is not unusual to discontinue a longitudinal and demanding trial because of other comorbidities such as age or health or family issues. Especially for these preliminary data with such a small cohort we did not want to waste the data of any participants. We annotated the number of participants contributing to each time point of data in the figures. We also verified that the dropouts were not for reasons related to the effects of the trial but rather to health and family reasons and, therefore, part of what is considered random variation for missing values in clinical trials.

We dealt explicitly with the issue of missing data in the analysis of results: if we had any missing data from any time point at any period of the stimulation condition, we discarded that period’s results from that participant, however, we kept this patient’s results for the other phase if they were available so we could increase our sample size. We made our calculations based on repeated measures within each phase of stimulation rather than grossly within each participant across both phases. However, in the analysis of results, we performed a model selection: if the data at any follow-up time point were significantly different between periods, the model would not combine the effects of both periods. In this way, the combined model was selected for trained (confirming the same direction of effects despite the missing data in the second period) and the interaction model was selected for the untrained.

## Conclusion

This study showed that tDCS when used in conjunction with SLT, improves written verb naming and spelling performance in PPA. The results of this study provide additional evidence for the use of tDCS alongside traditional language therapy in PPA to target verb-naming deficits. Due to the complexity of verbs, future studies should seek to determine the effects of tDCS on verbs at the sentence level.

## Ethics Statement

This study was approved by the Johns Hopkins Institutional Review Board. All subjects gave written informed consent in accordance with the Declaration of Helsinki.

## Author Contributions

KT and CF contributed to the conception and design. BF and KW acquired the data. BF, CF, AF, KW, and KT analyzed and interpreted the data. AF, KW, BF, CF, and KT wrote and revised the manuscript critically for intellectual content.

## Conflict of Interest Statement

The authors declare that the research was conducted in the absence of any commercial or financial relationships that could be construed as a potential conflict of interest.

## Footnotes

1 www.wongbakerfaces.org

2 http://wongbakerfaces.org/

## Supplementary Material

FIGURE S1 Percent gain in performance for trained items, combining both periods. Top and bottom rows of numbers represent the number of people in the sham and tDCS groups, respectively.

FIGURE S2 Percent gain in performance for untrained items, combining both periods. Top and bottom rows of numbers represent the number of people in the sham and tDCS groups, respectively.

DOCUMENT S1 Grapheme-by-grapheme scoring system used for calculation of written-naming scores.
