# Supplementary figures and images for: Written Verb Naming Improves After tDCS Over the Left IFG in Primary Progressive Aphasia
Source: Front Psychol. 2019 Jun 12;10:1396. doi: 10.3389/fpsyg.2019.01396 (PMC6582664; doi:10.3389/fpsyg.2019.01396)

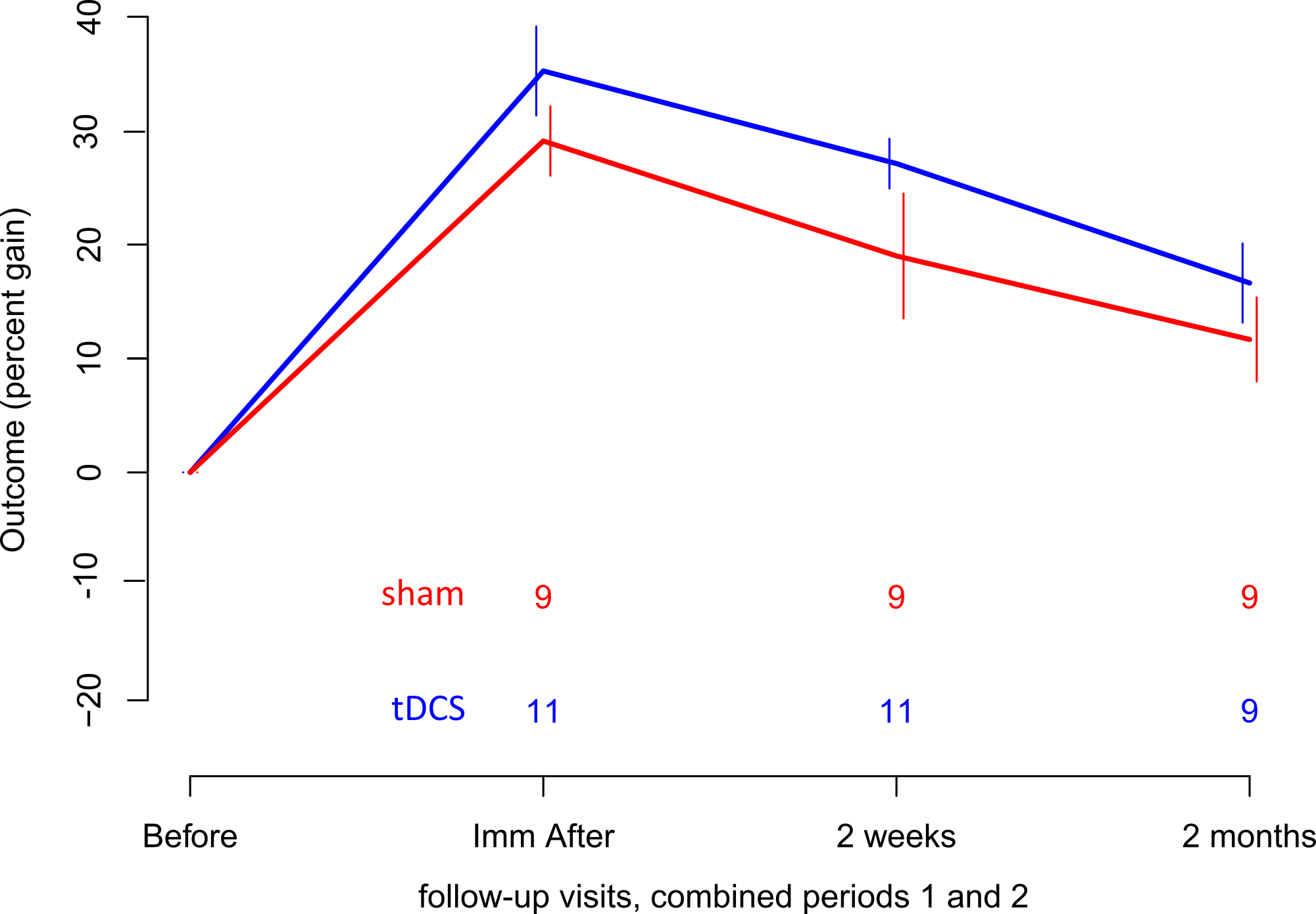

Supplement: FIGURE S1 — Percent gain in performance for trained items, combining both periods. Top and bottom rows of numbers represent the number of people in the sham and tDCS groups, respectively. [file Image_1.TIF]

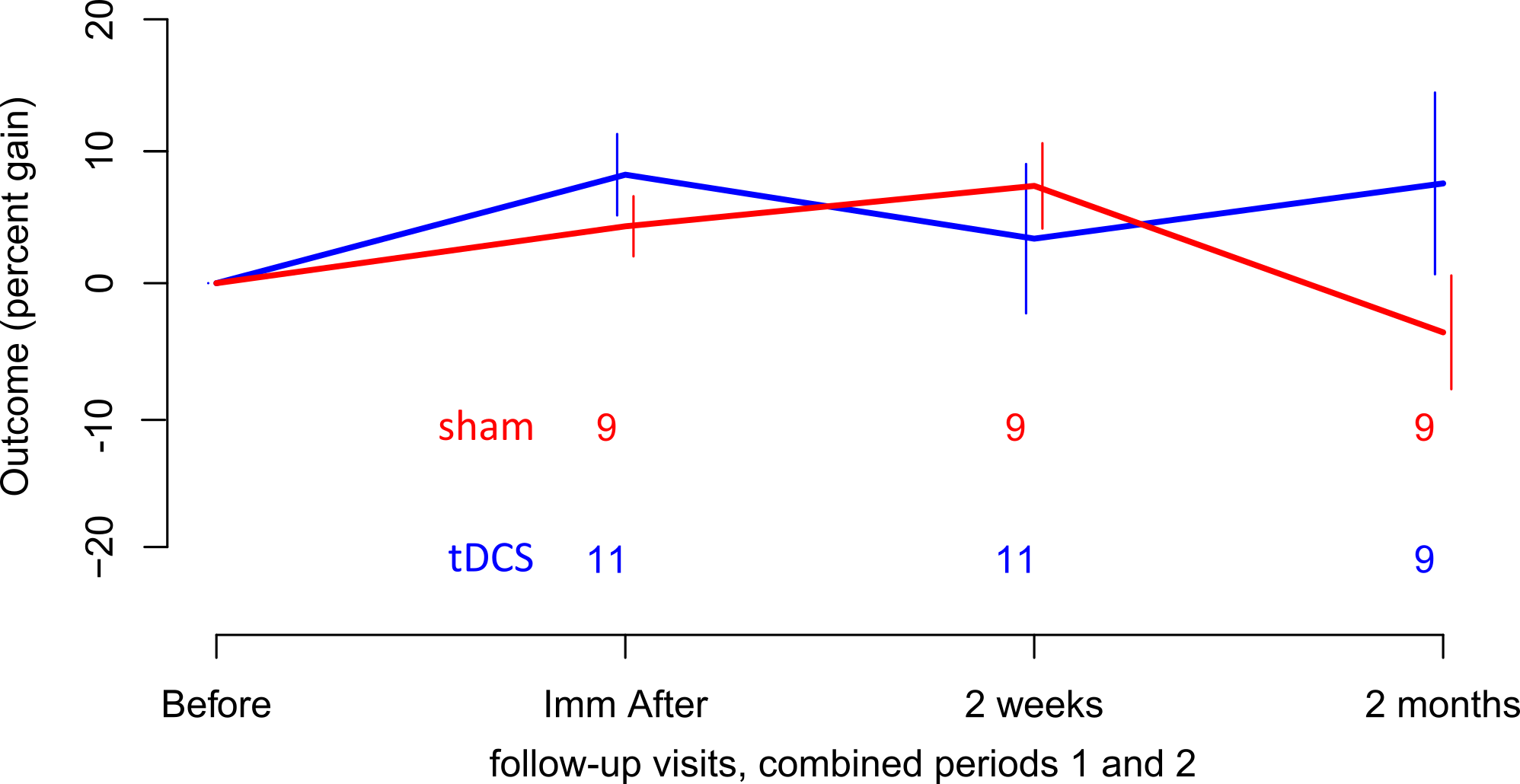

Supplement: FIGURE S2 — Percent gain in performance for untrained items, combining both periods. Top and bottom rows of numbers represent the number of people in the sham and tDCS groups, respectively. [file Image_2.TIF]
