# Supplementary material for: Written Verb Naming Improves After tDCS Over the Left IFG in Primary Progressive Aphasia
Source: Front Psychol. 2019 Jun 12;10:1396. doi: 10.3389/fpsyg.2019.01396 (PMC6582664; doi:10.3389/fpsyg.2019.01396)
Supplement: DOCUMENT S1 — Grapheme-by-grapheme scoring system used for calculation of written-naming scores. [file Data_Sheet_1.PDF]

Spelling Scoring System [Goodman and Caramazza, 1985]

DIRECTIONS FOR SCORING

EACH LETTER OF THE TARGET WORD IS ASSIGNED A VALUE {0,.5,1}
ANY LETTER WHICH IS PRESENT & IN THE CORRECT POSITION (RELATIVE, NOT ABSOLUTE) GETS 1 POINT
ANY LETTER WHICH IS PRESENT BUT IN THE INCORRECT POSITION GETS .5 POINTS
ANY LETTER WHICH IS NOT PRESENT GETS 0 POINTS

TO DETERMINE THE VALUE,

1. MAXIMALLY ALIGN THE TARGET WORD & THE RESPONSE
e.g.

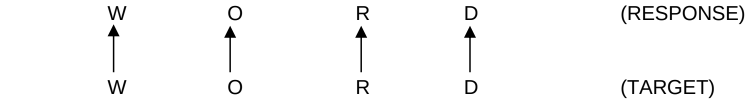

e.g.

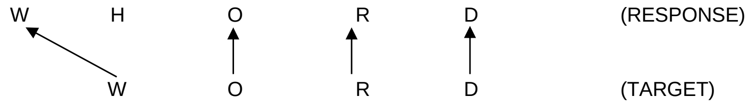

e.g.

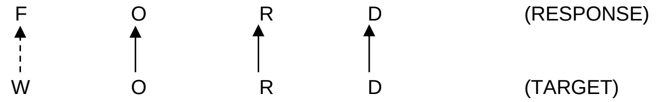

e.g.

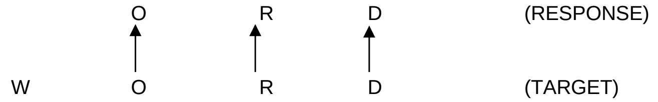

e.g.

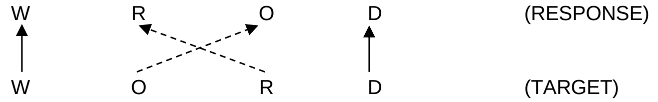

2. IF THE RESPONSE IS CORRECT, GIVE EACH TARGET LETTER VALUE 1

e.g.

W	O	R	D	(RESPONSE)
↑	↑	↑	↑	
W	O	R	D	(TARGET)
1	1	1	1	(SCORE)

3. IF THE RESPONSE IS INCORRECT, SCORE DEPENDING UPON ERROR TYPE.

3A. **ADDITION**-GIVE EACH OF THE LETTERS WHICH ALLOW THE ADDITION VALUE **.5**

e.g.

W	H	O	R	D	(RESPONSE)
↖		↑	↑	↑	
	W	O	R	D	(TARGET)
	0.5	0.5	1	1	(SCORE)

MARK THE ADDITION TYPE IN THE **NOTES** COLUMN.

A=ADDITION OF A LETTER NOT FOUND ELSEWHERE IN THE TARGET
- e.g. WORD-->WHORD **A(H)**
- e.g. WORD-->WHOERD **A(H), A(E)**

AP=ADDITION OF A LETTER FOUND EARLIER IN THE TARGET (PERSEVERATIVE)
- e.g. WORD-->WORWD **AP(W)**

AA=ADDITION OF A LETTER FOUND LATER IN THE TARGET (ANTICIPATORY)
- e.g. WORD-->WRORD **AA(R)**

3B. **SUBSTITUTION**-GIVE THE LETTER WHICH IS SUBSTITUTED FOR VALUE 0

MARK THE SUBSTITUTION TYPE IN THE **POSITION** COLUMN

S=SUBSTITUTION OF A LETTER NOT FOUND ELSEWHERE IN THE TARGET
- e.g. WORD-->FORD **S(W>F)**

SP=SUBSTITUTION OF A LETTER FOUND EARLIER IN THE TARGET (PERSEVERATIVE)
- e.g. WORD-->WORO **SP(D>O)**

SA=SUBSTITUTION OF A LETTER FOUND LATER IN THE TARGET (ANTICIPATORY)
- e.g. WORD-->DORD **SA(W>D)**

e.g.

F	O	R	D	(RESPONSE)
⇡	↑	↑	↑	
W	O	R	D	(TARGET)
S(W>F)	1	1	1	(SCORE)

3C. **DELETION**-GIVE THE LETTER WHICH IS DELETED VALUE 0

MARK **D** IN **POSITION** COLUMN

e.g.

	O	R	D	(RESPONSE)
W	O	R	D	(TARGET)
D	1	1	1	(SCORE)

3D. **TRANSPOSITION-**GIVE VALUE .5 TO EACH TRANSPOSED LETTER

e.g.

W	D	R	O	(RESPONSE)
W	O	R	D	(TARGET)
1	0.5	1	0.5	(SCORE)

3E. **MOVEMENT**-GIVE VALUE .5 TO THE MOVED LETTER

e.g.

W	D	O	R	(RESPONSE)
W	O	R	D	(TARGET)
1	1	1	0.5 (moving letter)	(SCORE)

3F. **DOUBLING-**GIVE VALUE .5 TO DOUBLED LETTER

e.g.

W	O	O	R	D	(RESPONSE)
	W	O	R	D	(TARGET)
	1	0.5	1	1	(SCORE)

MARK THE DOULBING IN THE **NOTES** COLLUMN
e.g. WORD-->WOORD **DBL(O)**

BUT COUNT AS **SA/P** WHEN POSSIBLE
e.g.

R	O	O	S	T	(RESPONSE)
R	O	A	S	T	(TARGET)
1	1	SP(A>O)	1	1	(SCORE)

3G. **DELETION OF DOUBLE LETTER**-GIVE VALUE .5 TO BOTH LETTERS OF DOUBLE

e.g.

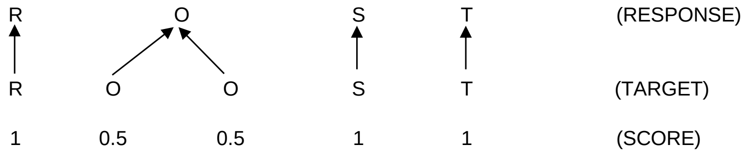

e.g.

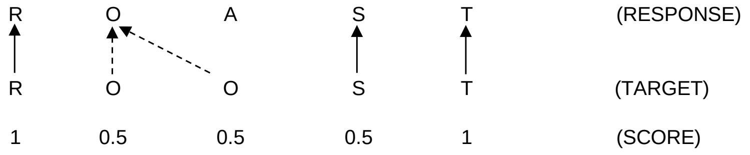

MARK ADDITION OF A IN NOTES

3H. **MOVEMENT OF GEMINATE**-

e.g.

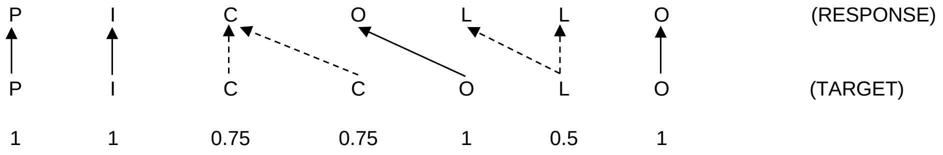

3I. **SUBSTITUTION/DELETION COMBINATION --** GIVE VALUE 0 TO MISSING LETTERS

MARK **S/D** IN THE **POSITION COLUMN**

e.g.

	A	R	D	(RESPONSE)
?	?	↑	↑	
W	O	R	D	(TARGET)
S/D	S/D	1	1	(SCORE)

MARK THE S/D EXAMPLE IN THE **NOTES** COLUMN. MARK Per() OR Ant() IF A LETTER IF PERSEVERATIVE OR ANTICIPATORY

e.g. WORD-->ARD **S/D(WO>A)**

e.g. WORD-->DRD **S/D(WO>D), Ant(D)**

3J. **SUBSTITUTION/ADDITION COMBINATION** - GIVE 0 TO INCORRECT LETTER, 0.75 TO SURROUNDING LETTERS

MARK **S/A** IN THE **POSITION** COLUMN

e.g.

W		A	I	R	D	(RESPONSE)
↖				↑	↑	
	W		O	R	D	(TARGET)
	0.75		S/A	0.75	1	(SCORE)

MARK S/A IN THE **NOTES** COLUMN. MARK Per() OR Ant() IF A LETTER IF PERSEVERATIVE OR ANTICIPATORY

e.g. WORD-->WAIRD **S/A(O>AI)**

e.g. WORD-->WOLWD **S/A(R>LW), Per(W)**

4. WHEN DEALING WITH MULTIPLE ERRORS IN A SINGLE RESPONSE,
DO NOT ASSUME MOVEMENT/TRANSPOSITION OF SUBSTITUTED LETTERS

e.g. THE RIGHT WAY think of the H as an addition that the W and O have 'allowed', and subtract .5 from the 'allowing' letters

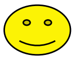

W	(H)	O	D	(RESPONSE)
↑	↗		↑	
W	O	R	D	(TARGET)
1	1	0	1	
0.5	0.5	D	1	(SCORE)

e.g. THE WRONG WAY

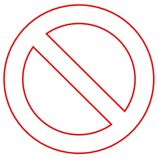

W	H	O	D	(RESPONSE)
↑	↗	↖	↑	
W	O	R	D	(TARGET)
1	0.5	s(r>h)	1	(SCORE)

TRANSPOSITION SUBSTITUTION
